# Diverse ‘just-right’ levels of chromosomal instability and their clinical implications in neoadjuvant treated gastric cancer

**DOI:** 10.1038/s41416-021-01587-4

**Published:** 2021-10-20

**Authors:** Meike Kohlruss, Marie Krenauer, Bianca Grosser, Nicole Pfarr, Moritz Jesinghaus, Julia Slotta-Huspenina, Alexander Novotny, Alexander Hapfelmeier, Thomas Schmidt, Katja Steiger, Matthias M. Gaida, Magdalena Reiche, Lukas Bauer, Katja Ott, Wilko Weichert, Gisela Keller

**Affiliations:** 1 Institute of Pathology, TUM School of Medicine, Technical University of Munich, Munich, Germany; 2 Institute of Pathology and Molecular Diagnostics, University Hospital Augsburg, Augsburg, Germany; 3 Institute of Pathology, University Hospital Marburg, Marburg, Germany; 4 Department of Surgery, TUM School of Medicine, Technical University of Munich, Munich, Germany; 5 Institute of Medical Informatics, Statistics and Epidemiology, Technical University of Munich, Munich, Germany; 6 Institute of General Practice and Health Services Research, TUM School of Medicine, Technical University of Munich, Munich, Germany; 7 Department of Surgery, University of Heidelberg, Heidelberg, Germany; 8 Department of Surgery, Universitätsklinikum Köln, Köln, Germany; 9 German Cancer Consortium (DKTK), Partner Site Munich, Institute of Pathology, Munich, Germany; 10 Institute of Pathology, University of Heidelberg, Heidelberg, Germany; 11 Institute of Pathology, University Medical Center Mainz, JGU-Mainz, Mainz, Germany; 12 Department of Surgery, RoMed Klinikum Rosenheim, Rosenheim, Germany

**Keywords:** Gastric cancer, Surgical oncology, Prognostic markers, Predictive markers

## Abstract

**Background:**

The Cancer Genome Atlas (TCGA) consortium described EBV positivity(+), high microsatellite instability (MSI-H), genomic stability (GS) and chromosomal instability (CIN) as molecular subtypes in gastric carcinomas (GC). We investigated the predictive and prognostic value of these subtypes with emphasis on CIN in the context of neoadjuvant chemotherapy (CTx) in GC.

**Methods:**

TCGA subgroups were determined for 612 resected adenocarcinomas of the stomach and gastro-oesophageal junction (291 without, 321 with CTx) and 143 biopsies before CTx.

EBV and MSI-H were analysed by standard assays. CIN was detected by multiplex PCRs analysing 22 microsatellite markers. Besides the TCGA classification, CIN was divided into four CIN-subgroups: low, moderate, substantial, high. Mutation profiling was performed for 52 tumours by next-generation sequencing.

**Results:**

EBV(+) (HR, 0.48; 95% CI, 0.23–1.02), MSI-H (HR, 0.56; 95% CI, 0.35–0.89) and GS (HR, 0.72; 95% CI, 0.45–1.13) were associated with increased survival compared to CIN in the resected tumours. Considering the extended CIN-classification, CIN-substantial was a negative prognostic factor in uni- and multivariable analysis in resected tumours with CTx (each *p* < 0.05). In biopsies before CTx, CIN-high predicted tumour regression (*p* = 0.026), but was not prognostically relevant.

**Conclusion:**

A refined CIN classification reveals tumours with different biological characteristics and potential clinical implications.

## Background

Chromosomal instability (CIN) is one of the major hallmarks of many solid tumours and is considered as a type of genomic instability driving the carcinogenic process by an ongoing acquisition of genomic alterations including gains or losses of whole chromosomes or only parts of them [1]. Based on a comprehensive genome-wide analysis performed by the Cancer Genome Atlas (TCGA) consortium [2], CIN, defined by the degree of chromosomal alterations rather than by a measured rate, emerged as the most common molecular subtype in gastric carcinomas (GC). Besides CIN, three other molecular subtypes encompassing Epstein-Barr virus positive (EBV+) tumours, tumours with high microsatellite instability (MSI-H) and genomic stable (GS) tumours were identified [2]. This categorisation is clinically relevant, with an association with better outcomes identified for EBV(+) and MSI subtypes in several studies [3–9], whereas the positive or predictive impact of CIN in GC is poorly characterised. This may in part be due to the lack of consistent techniques for the precise determination of CIN especially in the routine diagnostic setting and due to the lack of a standardised definition of CIN, while for the detection of EBV or MSI relatively simple diagnostic methods based on immunohistochemistry, in situ hybridisation or PCR and clear definitions exist [10, 11]. To overcome this restriction we had developed a cost-efficient and reliable microsatellite-based multiplex PCR assay for the detection of allelic imbalance (AI) as a surrogate measure for CIN in a previous study [12].

In this study, we first investigated the prognostic and predictive role of the molecular classification as proposed by the TCGA consortium in our large GC cohorts in the context of neoadjuvant chemotherapy (CTx) using this assay. In a second step we asked, if a further subdivision and characterisation of the CIN-group may reflect in more detail the biologically heterogeneous nature of GC and may specifically contribute to a more differentiated impact of CIN on response to CTx and prognosis of the patients.

## Methods

### Patient cohorts and study enrolment

Surgically resected specimens from 617 patients with gastric adenocarcinomas including tumours of the gastro-oesophageal junction (AEGII and AEGIII according to Siewert and Stein [13]) that were treated with or without neoadjuvant CTx between 2001 and 2013 at the Department of Surgery of the University of Heidelberg and between 2001 and 2012 at the Technical University of Munich were initially included in the study. For five cases, no CIN data were available and the final resected tumour cohort for molecular analysis of the four subgroups EBV(+), MSI-H, GS and CIN according to the TCGA classification consisted of 612 resected specimens. Tumour biopsies before neoadjuvant CTx from 143 patients treated between 1993 and 2013 at the Department of Surgery of the Technical University of Munich were also included. Characteristics of all patients included for molecular analysis were as described [3]. For 42 patients, corresponding pre-treatment biopsies before CTx and post-treatment resected specimens after CTx were available to determine tumour cell plasticity of the molecular subgroups in the context of neoadjuvant CTx. Limitation for inclusion into the molecular analysis were the availability of paraffin blocks and sufficient DNA from tumour and non-tumorous tissues.

**Fig. 1:**
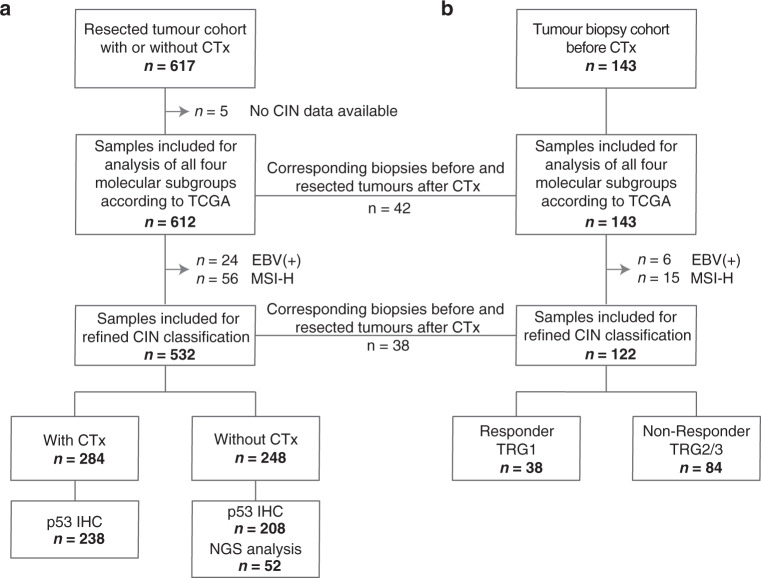
Flow diagram of patient and specimen inclusion. The total number of included patients for molecular classification in the four subgroups EBV(+), MSI-H, GS and CIN according to TCGA, for the refined CIN classification and for the additional p53 IHC and NGS analysis is shown for the resected tumour cohort with or without CTx (**a**) and the tumour biopsy cohort before CTx (**b**). MSI-H high microsatellite instability, GS genomic stable, CIN chromosomal instability, TRG tumour regression grade, IHC immunohistochemistry, NGS next-generation sequencing.

The refined CIN classification was performed only for EBV and MSI-H negative tumours and these final cohorts consisted of 532 patients with resected tumours (284 with CTx and 248 without CTx) and of 122 patients with tumour biopsies before CTx. Patient characteristics for the refined CIN analysis are summarised in Table 1 and an overview of the study enrolment is shown in Fig. 1.

**Table 1:** Patient characteristics for the refined CIN classification.

		Resected tumour cohort with CTx	Resected tumour cohort without CTx	Tumour biopsy cohort before CTx
Category	Value	*n* (%)	*n* (%)	*n* (%)
Cases	Total	284 (100)	248 (100)	122 (100)
Age [yr]	Median	61.2	66.9	60.6
	Range	28.3–81.2	32.1–90.9	23.1–78.0
Overall survival [mo]	Median	30.3	61.1	44.6^a^
	95% CI	25.2–35.4	27.5–94.7	18.5–70.8
Follow-up period [mo]	Median	60.7	56.4	70.8
	95% CI	51.8–69.5	50.7-62.1	64.6–77.0
Sex	Male	226 (79.6)	165 (66.5)	92 (75.4)
	Female	58 (20.4)	83 (33.5)	30 (24.6)
Tumour localisation	Proximal	187 (65.8)	81 (32.7)	88 (72.1)
	Middle	52 (18.3)	74 (29.8)	17 (13.9)
	Distal	31 (10.9)	76 (30.6)	12 (9.8)
	Total/linitis	14 (4.9)	13 (5.2)	5 (4.1)
	n/a	0	4 (1.6)	0
Laurén classification	Intestinal	165 (58.1)	127 (51.2)	62 (50.8)
	Non-intestinal	119 (41.9)	121 (49.8)	60 (49.2)
Tumour grade	G1/2	42 (14.8)	71 (28.6)	30 (24.6)
	G3/4	167 (58.8)	176 (71.0)	92 (75.4)
	n/a	75 (26.4)	1 (<1)	0
Clinical tumour stage (cT)	cT2	14 (4.9)	112 (45.2)	7 (5.7)
	cT3/4	269 (94.8)	135 (54.4)	112 (91.8)
	n/a	1 (<1)	1 (<1)	3 (2.5)
(y)pT^b^	(y)pT0	0	0	8 (6.6)
	(y)pT1	12 (4.2)	38 (15.3)	10 (8.2)
	(y)pT2	26 (9.2)	42 (16.9)	17 (13.9)
	(y)pT3	166 (58.5)	113 (45.6)	69 (56.6)
	(y)pT4	80 (28.2)	55 (22.2)	16 (13.1)
	n/a	0	0	2 (1.6)
(y)pN^b^	Negative	71 (25)	87 (35.1)	53 (43.4)
	Positive	213 (75)	161 (64.9)	67 (54.9)
	n/a	0	0	2 (1.6)
Metastasis status	No	224 (78.9)	229 (92.3)	82 (67.2)
	Yes	60 (21.1)	19 (7.7)	38 (31.1)
	n/a	0	0	2 (1.6)
Resection category	R0	200 (70.4)	198 (79.8)	100 (82)
	R1	84 (29.6)	50 (20.2)	20 (16.4)
	n/a	0	0	2 (1.6)
Tumour regression grade	TRG1	–	–	38 (31.2)
	TRG2	141 (49.6)	–	33 (27.0)
	TRG3	143 (50.4)	–	51 (41.8)

*CTx* neoadjuvant chemotherapy, *CI* confidence interval, *MSI* microsatellite instability, *n/a* not available.

^a^OS was defined as time between the date of operation and death by any cause. For two patients who were not operated, the date of start of CTx was used.

^b^TNM classification according to 7th Edition UICC.

### Chemotherapy and surgery

Patients were treated with platinum/5-fluorouracil (5-FU) based chemotherapeutic regimens with or without taxane or anthracycline. The detailed chemotherapeutic regimens of the patients included for the refined CIN classification are shown in Supplementary Table S1. The surgical approaches included an abdominal D2 lymphadenectomy as reported in detail elsewhere [3, 14].

### Response evaluation

Response to preoperative CTx was determined histopathologically and classified into three tumour regression grades (TRG) according to the Becker classification: [15] TRG1, TRG2 and TRG3, which corresponded to <10%, 10–50% and >50% residual tumour cells present in the tumour bed after neoadjuvant CTx, respectively.

In the cohort of patients with tumour biopsies before CTx, all three regression grades were present within the resected specimen. Overall survival (OS) of the TRG1, TRG2 and TRG3 group was significantly different (*p* < 0.001) (Supplementary Fig. S1a). Comparing OS only between the patients with TRG2 and TRG3, no significant difference was observed (*p* = 0.344). Thus, for the purpose of this study, we combined TRG2 and TRG3 into one group and refer to them as the non-responding patients. Comparison of OS between the responding (TRG1 group) and the non-responding group was statistically significant (*p* < 0.001) (Supplementary Fig. S1b). In the resected tumour cohort with CTx, TRG1 tumours were not included either due to the complete absence of tumour cells or very low tumour cell contents which makes a molecular tumour analysis from these samples difficult or impossible.

### Follow-up and overall survival

Follow-up was performed as described and median follow-up was calculated by the inverse Kaplan–Meier method [14, 16]. OS of the patients was defined as the time between the date of surgery and death by any cause [3, 14]. Median follow-up and OS data of the patients included for the refined CIN analysis are shown in Table 1.

### DNA isolation and tumour cell content

DNA isolation from paired tumour and non-tumorous formalin-fixed paraffin-embedded (FFPE) tissues is described in Supplementary Material (Supplementary Methods).

Samples with a tumour cell content of at least 10% were included for MSI analysis according to the described limit of detection for MSI [17]. We investigated the AI ratios for an association with the tumour cell content divided into four groups in a range from 10 to 25%, 25 to 50%, 50 to 75% and ≥75% in all tumours used for MSI analysis. As no essential correlation of the AI ratios with tumour cell content was found (Pearson correlation coefficient r = 0.207), all samples were included in this study. The distribution of the AI ratios in association with the tumour cell content is shown in Supplementary Fig. S2.

### EBV and MSI analysis

EBV analysis was performed by a PCR-based assay and tumours with positive PCR signals were further analysed by chromogenic in situ hybridisation as described [3, 11].

MSI was analysed using the Bethesda panel consisting of the two mononucleotide repeats BAT25 and BAT26 and the three dinucleotide repeats D2S123, D5S346 and D17S250 as recommended by the National Cancer Institute [10] and is described in Supplementary Material (Supplementary Methods). According to a standardised definition, high MSI (-H) was defined if at least two of the five markers showed instabilities and as microsatellite stable (MSS) and/or low MSI (-L), if none or one of the five markers showed MSI, respectively. To avoid a classification as MSI-H based on instabilities exclusively at two dinucleotide repeats, those tumours were additionally analysed using the three mononucleotide markers NR-21, NR-24 and NR-27 as recommended [18]. If no mononucleotide marker was unstable, these tumours were classified as MSI-L. MSI analysis using the Bethesda panel and the additional three mononucleotide repeats are described in detail in Supplementary Material (Supplementary Methods). According to TCGA, tumours were classified into EBV(+), MSI-H and MSS/MSI-L tumours [2].

### Detection and classification of CIN

Allelic imbalance (AI) was detected as a surrogate measurement for CIN using a microsatellite based multiplex PCR assay consisting of 19 microsatellite markers covering 14 chromosomal regions [12]. The three dinucleotide repeats (D2S123, D5S346, D17S250) included in the standard NCI Bethesda-panel [10] used for the detection of MSI were also evaluated for CIN, thus a total of 22 microsatellite markers was analysed. Amplification of the fluorescence-tagged primers was performed in four multiplex PCRs using the Type-it Microsatellite PCR kit (Qiagen, Hilden, Germany). The analysed microsatellite markers, the cycle conditions and fragment analysis of the PCR products are described in Supplementary Material (Supplementary Methods and Supplementary Table S2). AI ratios per tumour were calculated by dividing the number of heterozygous markers having AI by the total number of informative markers resulting in AI values ranging from 0 to 1. The tumours were classified as CIN and GS essentially according to the definition of TCGA using a corresponding threshold of an AI ratio ≥0.2 for the classification of CIN and <0.2 of GS [12]. For a refined classification of CIN, the tumours were then subdivided into four pre-defined groups based on the calculated quartiles of the AI ratios per tumour. The particular CIN-groups were termed as follows: CIN-low (≤25%), CIN-moderate (>25% and ≤50%), CIN-substantial (>50% and <75%) and CIN-high (≥75%).

### Mutation profiling using next-generation sequencing

Mutation analysis was performed by targeted sequencing using the Ion Torrent platform (Thermo Fisher Scientific, Waltham, MA, USA) and a custom-designed sequencing gene panel (Ion AmpliSeq, Thermo Fisher Scientific) encompassing 525 amplicons covering coding regions of 58 GC related genes. The multiplex PCR based Ion AmpliSeq targeted sequencing technology (Thermo Fisher Scientific) was used for DNA library preparation and amplification of target regions using the Ion AmpliSeq Library Kit v2.0, as well as the specific GC sequencing panel consisting of four primer pools as described in detail previously [19, 20]. Automated template preparation of the final libraries as well as chip loading (Ion 520, 530, or 540) was performed on an Ion Chef instrument and sequenced using an Ion S5XL instrument (Thermo Fisher Scientific). Data analysis was performed referring to Pfarr et al. [20] and ANNOVAR was used to annotate the sequence variants [21].

### Expression analysis of p53

Immunohistochemical analysis of p53 expression had been performed previously and expression of p53 wild-type was defined as less than 60% tumour cells displaying nuclear p53 staining with variable intensity and aberrant p53 was present as either a complete loss of expression with 0% nuclear staining of tumour cells or an overexpression of p53 with more than 60% nuclear staining of tumour cells with medium to high intensity [19, 22].

### Statistical analysis

The statistical analyses are described in detail in Supplementary Material (Supplementary Methods).

## Results

### Frequency of the molecular TCGA subgroups

In a first step, the tumours were classified into the four molecular subgroups in analogy to the molecular TCGA classification system [2]. In the resected tumour cohort with or without CTx, 24 (3.9%) of the 612 tumours were EBV(+), 56 (9.2%) were MSI-H, 56 (9.2%) were GS and 476 (77.7%) were CIN. In the tumour biopsy cohort before CTx, 6 (4.2%) of the 143 biopsies were classified as EBV(+), 15 (10.5%) as MSI-H, 7 (4.9%) as GS and 115 (80.4%) as CIN. Results are included in Supplementary Table S3. Out of the overall 30 EBV-associated tumours, 5 (16.7%) were classified as GS and 25 (83.3%) as CIN. All MSI-H tumours were negative for EBV. MSI-H tumours were not evaluable for CIN due to numerous additional microsatellite alleles in the tumour, which does not allow a reliable evaluation of AI. The MSI status of the 42 paired biopsies before CTx and resected tumours after CTx were the same in all cases. None of these pairs was EBV(+).

### Molecular classification according to TCGA—prognostic and predictive relevance of the TCGA subgroups

**Fig. 2:**
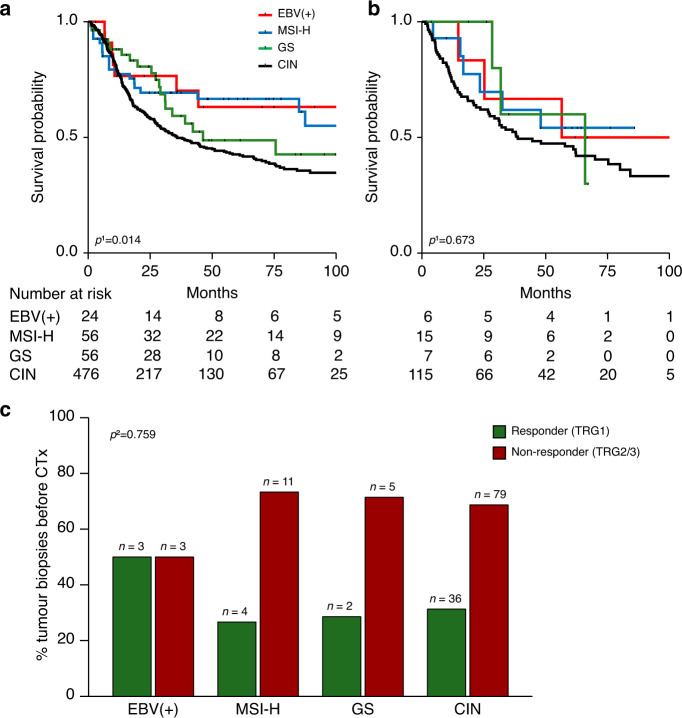
Molecular classification according to TCGA and association with patients’ survival and response to neoadjuvant CTx. Patients’ survival is discriminated by the four molecular TCGA subgroups and Kaplan–Meier curves are shown for patients with resected tumours with or without CTx (**a**) and for patients with tumour biopsies before CTx (**b**). The response to neoadjuvant CTx (**c**) is shown for patients with tumour biopsies before CTx in relation to the respective molecular subgroups. MSI-H high microsatellite instability, GS genomic stable, CIN chromosomal instability, TRG tumour regression grade. ^1^Cox’s regression; ^2^Fisher’s exact test (overall).

In the resected tumour cohort with or without CTx a prognostic relevance of the four molecular TCGA subgroups was indicated (overall *p* = 0.014, Fig. 2a). Patients with EBV(+) (HR, 0.48; 95% CI, 0.23–1.02; *p* = 0.056) and MSI-H (HR, 0.56; 95% CI, 0.35–0.89; *p* = 0.014) tumours showed the best survival followed by the GS group (HR, 0.72; 95% CI, 0.45–1.13; *p* = 0.149) compared to the CIN-group taken as reference. In the tumour biopsy cohort before CTx, no statistically significant difference was found in respect to survival of the patients (overall *p* = 0.673, Fig. 2b) and the TCGA classification comprising four subgroups was also not predictive for response to platinum/5-FU based CTx (overall *p* = 0.759, Fig. 2c).

### Refinement of CIN classification and prevalence of CIN-subgroups

As CIN was the most frequent subgroup (77.7–80.4%) in the resected tumour and the tumour biopsy cohort, we investigated if further subdivision of CIN in the EBV and MSI-H negative tumours could delineate distinct groups of patients with additional clinical characteristics. Thus, CIN was categorised into four pre-defined subgroups: low, moderate, substantial, high.

In the resected tumour cohort with or without CTx, 97 (18.2%) of the 532 tumours were classified as CIN-low, 214 (40.2%) as CIN-moderate, 161 (30.3%) as CIN-substantial and 60 (11.3%) as CIN-high. Regarding the frequencies of the CIN-subgroups in the tumour biopsy cohort before CTx, 10 (8.2%) of the 122 biopsies were classified as CIN-low, 40 (32.8%) as CIN-moderate, 48 (39.3%) as CIN-substantial and 24 (19.7%) as CIN-high. Results are included in Supplementary Table S3.

### Comparison of refined CIN classification in corresponding pre-therapeutic biopsies and post-therapeutic resected tumours

Comparison of the corresponding biopsies before CTx and resected tumours after CTx of overall 38 patients revealed a consistent classification in the respective CIN-groups in 10 (26%) cases and a different CIN classification in 28 (73%) cases. The alterations between the paired tumour specimens showed that 9 (32%) biopsies before CTx changed from a lower CIN status into a higher one in the corresponding resected specimen and 19 (68%) of the pre-treatment biopsies changed from a higher CIN subgroup into a lower one (Supplementary Fig. S3). Previously we showed, that the probability of a change of a specific CIN-group due to intra-tumour variability was only in the range of 10% [12]. Thus, this indicates that the majority of the observed alterations in the 38 paired tumour specimen in this study seems to be most likely related to the applied chemotherapy. Given these relevant differences in the comparison between paired biopsies before and resected tumours after CTx, subsequent analysis for prognostic and predictive relevance of the various CIN-subgroups was performed separately in the tumour biopsies before CTx, the resected tumours with CTx and the resected tumours without CTx.

### Refined CIN classification—frequency and association with clinical characteristics in the resected tumour cohort with and without CTx

Frequencies of the refined CIN-groups and association with clinical characteristics were analysed for the 284 patients with resected tumours with CTx and the 248 patients with resected tumours without CTx. In the resected tumour cohort with CTx, 18% of the tumours were classified as CIN-low, 42.6% as CIN-moderate, 28.5% as CIN-substantial and 10.9% as CIN-high. Similar frequencies of the subgroups were found in the resected tumours without CTx with 18.5% for CIN-low, 37.5% for CIN-moderate, 32.3% for CIN-substantial and 11.7% for CIN-high. Results are included in Supplementary Table S3.

Significant associations with tumour localisation and Laurén subtypes were evident in both cohorts (each *p* < 0.05). In the resected tumours without CTx, CIN-low was associated with poor differentiation (*p* = 0.001) and a lower clinical tumour stage cT2 (*p* = 0.022). Results are shown in Supplementary Tables S4 and S5.

### Refined CIN classification—prognostic relevance in the resected tumour cohort with and without CTx

**Fig. 3:**
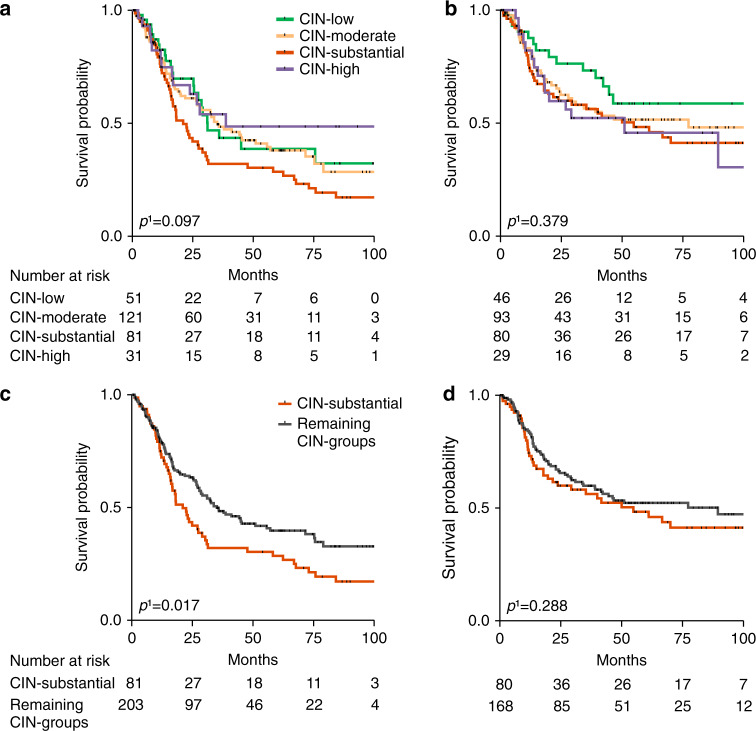
Refined CIN classification and association with patients’ survival in the resected tumour cohort with and without CTx. Kaplan–Meier curves are shown for patients with resected tumours with CTx regarding the four (**a**) and two refined CIN-subgroups (**c**) and for patients with resected tumours without CTx regarding the four (**b**) and two refined CIN-subgroups (**d**). CIN chromosomal instability. ^1^Cox’s regression.

In the resected tumours with or without CTx, no significant differences of the four CIN-subgroups regarding OS were observed, but particularly among the resected tumours with CTx distinctive features were apparent (overall *p* = 0.097) (Fig. 3a, b). Patients harbouring tumours with high levels of CIN (>75%) followed by the CIN-moderate and CIN-low group revealed the best survival whereas the CIN-substantial (third quartile) group obviously showed the worst survival in this tumour cohort (Fig. 3a). In the resected tumours without CTx, the CIN-low subgroup revealed the best survival compared to the other CIN-groups although the difference was not statistically significant (overall *p* = 0.379, Fig. 3b). All survival data of the different patient cohorts are summarised in detail in Supplementary Table S6.

Comparing the CIN-substantial group with the remaining CIN-groups revealed a statistically significant difference particularly in the resected tumour cohort with CTx (HR 1.49, 95% CI 1.07-2.08; *p* = 0.017, Fig. 3c), but not for the resected tumour cohort without CTx (HR 1.24, 95% CI 0.84-1.83; *p* = 0.288; Fig. 3d) (Supplementary Table S6).

To analyse if the decreased OS of the CIN-substantial tumours may be related to a preponderance of proximally located tumours or of clinically advanced tumour stages among the resected tumours with CTx, respective subgroup analyses were performed. Stratifying the patients according to tumour localisation revealed that the CIN-substantial group showed obviously the worst survival in patients with proximal and also in non-proximal located tumours in the resected tumour cohort with CTx (Supplementary Fig. S4a, b) whereas in the resected tumour cohort without CTx again no significant differences were observed (Supplementary Fig. S4c, d). Subgroup analysis within the resected tumour cohort without CTx stratified according to cT revealed also no significant differences in OS regarding the CIN-substantial group when compared to the remaining CIN-groups (Supplementary Fig. S4e, f).

### Refined CIN classification—multivariable analysis in the resected tumour cohort with and without CTx

In multivariable analysis including the CIN status (CIN-substantial vs remaining CIN-groups) as a factor and adjusting for pre-therapeutically available clinical factors in the resected tumour cohort with CTx, the two-tiered CIN status was a significant prognostic factor (*p* = 0.019). Including the post-therapeutically available factors, the two-tiered CIN-status (*p* = 0.026) also emerged as a significant independent prognostic factor along with R-category (*p* = 0.001), (y)pN (*p* < 0.001), (y)pT (*p* = 0.003) and M-category (*p* = 0.002) in the resected tumours with CTx (Supplementary Table S7). In multivariable analysis of the resected tumour cohort without CTx, only the pre-therapeutically clinical variables cT and age (each *p* < 0.001) and the post-therapeutically variables pN (*p* < 0.001), age (*p* = 0.002), M-category (*p* = 0.010) and localisation (*p* = 0.025) emerged as prognostic relevant factors (Supplementary Table S8).

### Refined CIN classification—predictive and prognostic relevance in the tumour biopsy cohort before CTx

**Fig. 4:**
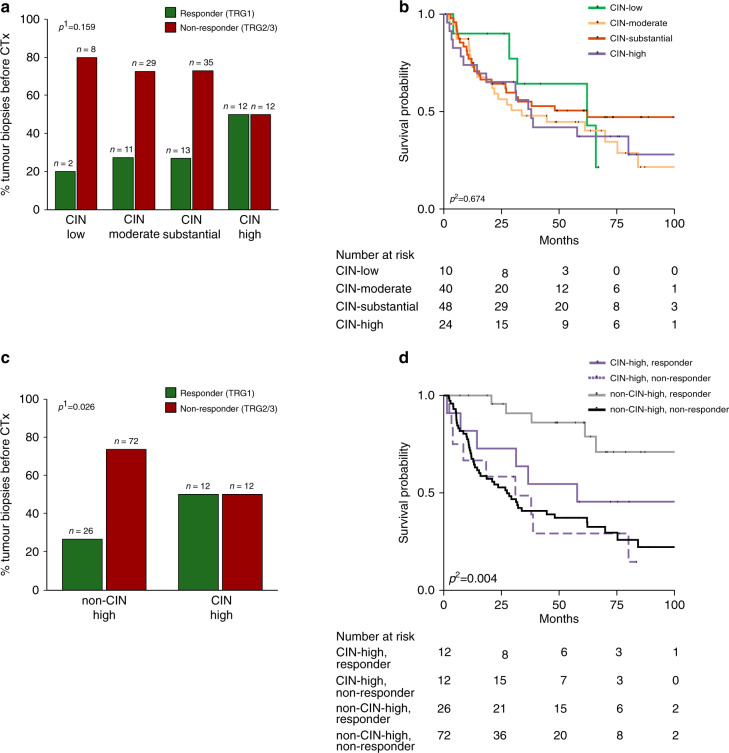
Refined CIN classification and association with response to neoadjuvant CTx and patients survival in the tumour biopsy cohort before CTx. The response to neoadjuvant CTx (**a**, **c**) and association with survival (**b**, **d**) is shown for patients with tumour biopsies before CTx in relation to the respective CIN-subgroup. CIN chromosomal instability, TRG tumour regression grade, ^1^Chi-squared test (overall). ^2^Cox’s regression.

Response to neoadjuvant CTx in terms of measureable tumour regression was analysed in the 122 tumour biopsies before CTx. No significant association with response (overall *p* = 0.159, Fig. 4a) nor with survival of the patients was found considering the four CIN-groups separately (overall *p* = 0.674, Fig. 4b).

However, the CIN-high group (>75%) when compared to the remaining CIN-groups demonstrated a statistically significant association with good response (*p* = 0.026, Fig. 4c) although this did not directly translate into differences in survival of the patients.

Analysis of this two CIN-groups regarding OS in the responding and non-responding patients separately, showed significant differences (overall *p* = 0.004, Fig. 4d). Interestingly however, non-CIN-high responding tumours showed the best survival (HR, 0.19; 95% CI, 0.08–0.48; *p* < 0.001) followed by the CIN-high responding (HR, 0.62; 95% CI, 0.26-1.44; *p* = 0.226) and the CIN-high non-responding group (HR, 1.10; 95% CI, 0.54-2.26; *p* = 0.786) taken the non-CIN-high non-responding group as reference. Survival data are included in Supplementary Table S9.

### Molecular characterisation of the refined CIN-groups

**Fig. 5:**
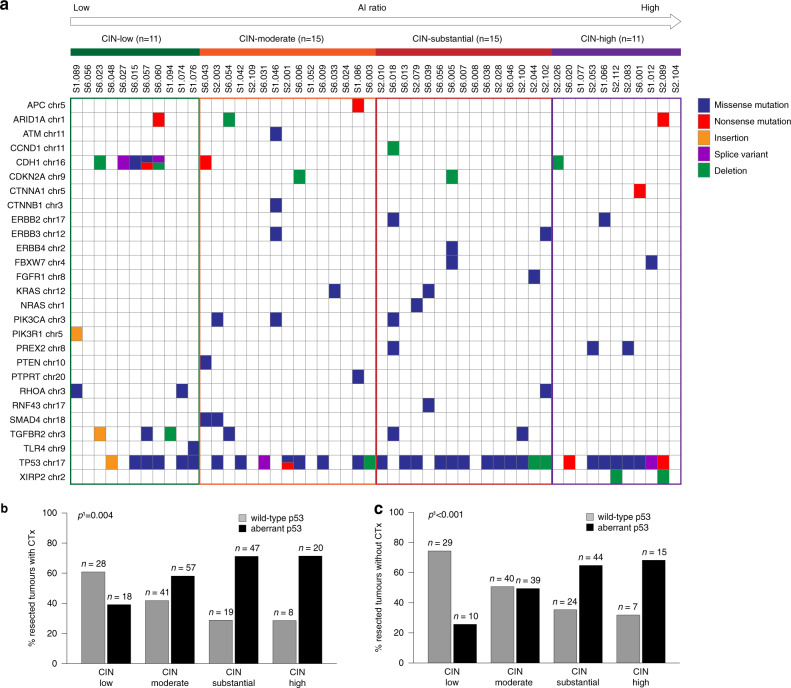
Molecular characteristics of the four refined CIN-groups. Mutational spectra by next-generation sequencing were determined for a subset of 52 tumours (**a**) in relation to the four refined CIN-groups. Expression analysis of p53 is shown for 238 resected tumours with CTx (**b**) and 208 resected tumours without CTx (**c**). AI allelic imbalance, CIN chromosomal instability. ^1^Chi-squared test (overall).

Due to the prognostic impact of the specific CIN-groups, we performed a more detailed molecular characterisation of these groups by targeted sequencing of 52 tumours using a GC related gene panel. All 52 tumours were from patients treated without CTx and were selected according to a relatively balanced distribution in the four refined CIN-groups. Accordingly, 11 (21%) of these 52 tumours were classified as CIN-low, 15 (29%) as CIN-moderate, 15 (29%) as CIN-substantial and 11 (21%) as CIN-high. Overall, the most frequent sequence variants were identified in *TP53* (65.4%) followed by *CDH1* (15.4%) and *TGFBR2* (11.5%). Five (71%) of the seven tumours with a *CDH1* mutation were classified as CIN-low and frequency of *TP53* mutations increased from lower CIN levels to higher ones from 28.5% (CIN-low) to 37.5% (CIN-moderate) and 40% (CIN-substantial) to 47% (CIN-high). Mutations in genes that were linked to the PI3K/AKT-signalling like the ERBB or RAS family of proteins or in the gene *PIK3CA* tended to be more frequent in the CIN-substantial group (Fig. 5a). All detected sequence variants are listed in detail in Supplementary Table S10.

For further characterisation of the various CIN-groups, an immunohistochemical analysis of p53 expression was evaluated for 238 resected tumours with CTx and for 208 resected tumours without CTx. Higher levels of CIN (>50%) were significantly associated with aberrant p53 expression in the resected tumours with and without CTx (*p* = 0.004 and *p* < 0.001, respectively) (Fig. 5b, c).

## Discussion

In this study, we performed a comprehensive molecular analysis to investigate the prognostic and predictive value of the molecular classification system according to TCGA in gastric/gastroesophageal adenocarcinomas. In addition, we were particularly interested in CIN and applied a refined, more detailed classification of this molecular subgroup. In line with the definition of CIN by the TCGA consortium, the term CIN as we used it in our study, refers to the description of a status of chromosomal abnormalities reflected by allelic imbalances suggestive of CIN and not to an ongoing acquisition of genomic alterations as has been suggested by others [1, 23].

In our study, the molecular classification according to TCGA was not predictive for response to a platinum/5-FU based CTx. A prognostic relevance, however, was found in the resected tumour cohort with or without CTx with the MSI-H and EBV(+) group showing essentially the best survival of the patients and the CIN-group the worst. These findings are in line with previous reports and underline the prognostic impact of MSI-H and EBV positive tumours [2, 3, 7, 9, 24].

As the CIN-group represents with 78–80% by far the most frequent molecular subtype in the resected tumours with or without CTx and the tumour biopsies before CTx, respectively, we investigated a refined CIN classification for the clinical relevance of specific CIN-subtypes.

In respect to this refined CIN classification, the most interesting results were first, the prognostic impact specifically for the CIN-substantial group in the resected tumours with CTx, which indicated worse survival and second the impact of CIN-high in the tumour biopsies before CTx, which predicted good response in terms of measurable tumour regression. Of note, however, this did not translate into a considerable survival benefit of the patients.

A prognostic relevance of CIN has been demonstrated in numerous studies and a high level of CIN was preferentially associated with aggressive tumour growth, tumour progression and metastasis in several tumour entities, although controversial results exist [25–31].

In our study, the CIN-substantial group corresponding to AI ratios in the third quartile, and not the group with the highest CIN degree, showed this significant association with worse survival. This finding is well in line with some studies that proposed a ‘just-right’ model of CIN presuming that only an optimal level of CIN can lead to tumour progression [32–34]. Moreover, a paradoxical non-monotonic relationship of CIN and prognosis was reported in GCs and breast carcinomas [30, 35]. Further studies using a specific CIN expression signature as a surrogate for CIN revealed an association of an intermediate expression level with the worst prognosis in breast carcinomas and also in a limited cohort of gastric cancer patients [35]. On the other side, an association of extreme CIN with improved outcome has been reported in oestrogen negative breast cancer [30]. Thus, our results are supporting the hypothesis that there is an optimal range of CIN promoting tumour growth, but there is a limit of the tumours ability to tolerate extensive chromosomal alterations, and when exceeding a specific threshold cell viability will decrease. Interestingly, if more than 75% of a cancer genome is affected by single copy number alterations, this threshold has been observed to be critical in this context in several tumour entities [33]. We observed the specific prognostic impact of the CIN-substantial group specifically for patients with resected tumours after CTx. We wondered if this might be related to the preponderance of patients with advanced tumour stages and proximally located tumours in the resected tumour cohort with CTx and we performed an analysis in the respective subgroup with and without CTx. There were no obvious differences in respect to tumour localisation, but a somewhat worse outcome of the CIN-substantial group was observed for patients with advanced cT3/cT4 also in the resected tumours without CTx. However, the negative prognostic impact seems to be more critically after treatment with CTx and the CIN-substantial group emerged also as an independent prognostic factor in multivariable analysis in this cohort. It is known that chemotherapy may exert a number of effects on the tumour cells themselves and also on the tumour microenvironment, which may lead to the induction of immunosuppression or an augmentation of anti-tumour immunity [36–38]. The interactions between the tumour cells and the immune cells are highly complex and they may lead to different biological behaviours of the tumours despite demonstrating the same CIN-groups but treated either with or without neoadjuvant CTx. Alternatively, CTx may induce or select a more aggressive subclone of the tumour cells specifically in the CIN-substantial group. From the clinical point of view, determination of CIN in resected tumours after CTx might be useful for the implementation of refined adjuvant treatment protocols or specific second line therapies.

The second important result of our study was the significant association of the CIN-high group with good response in terms of measurable tumour regression to a platinum/5-FU based neoadjuvant CTx. This again may be in accordance with a just-right concept of CIN for optimal tumour growth and indicates that when CIN exceeds a certain level, the tumour does not tolerate additional genetic alterations induced by DNA damaging agents and thus becomes more sensitive to them.

The comparison of the CIN levels between tumour biopsies before CTx and the corresponding resected tumours after CTx showed that 50% of the analysed paired samples changed from a higher CIN-level in the biopsy to a lower CIN state in the resected specimen after CTx. This could be due to an increased sensitivity of tumour cells with a higher degree of CIN to platinum/5-FU based CTx, which might further support this concept. Thus, essentially as suggested by others, a high degree of chromosomal instability may represent an ‘Achilles heel’ when the tumour is attacked by the right agents [33, 39, 40]. Interestingly, however, the association of CIN-high indicating a good response in terms of tumour regression did not translate into a significant survival benefit of the patients. One hypothesis might be that due to a preferentially targeting of CIN-high tumour cells by the applied CTx, a tumour subclone with a lower ‘just-right’ CIN level for tumour progression might be selected.

Another possibility might be again, that the intrinsic properties of the tumour cells together with the immune system may play a critical role and the determination of the CIN status represents just one aspect in this intricate biological scenario. Complex interactions between chemotherapy-induced cell death with the tumour microenvironment and immune components are supposed to contribute to therapeutic success [41, 42] and a considerable diversity of immunophenotypes of gastro-oesophageal adenocarcinomas have been described [38, 43, 44].

Of note, high levels of single copy number alterations were specifically associated with markers of immune evasion in various tumour types and immunological ‘cold’ tumours are usually associated with impaired prognosis [37, 45]. Thus, it is tempting to hypothesise that the lack of a substantial increased survival of the patients with CIN-high tumours despite a considerable tumour regression after CTx in our study may be related to the property of an immunological cold tumour counteracting a potential benefit due to tumour shrinkage. This apparently paradox situation of a good response of the tumour cells to neoadjuvant CTx, but without considerable survival benefit of the patients, may be clinically relevant for a prognostic differentiation of the subgroup of patients with complete or nearly complete tumour regression after neoadjuvant CTx. The combined consideration of the CIN status in the tumour biopsy before CTx and the tumour regression grade after CTx showed that specifically patients with non-CIN-high in the biopsy and TRG1 after neoadjuvant CTx have the best prognosis at all.

Regarding an association with other clinical characteristics of the patients, an increased level of CIN was significantly associated with proximal tumour location and intestinal tumour type in our study, which is in line with previous reports [2, 46] and emphasises the molecular heterogeneity of GC and differences in cancer biology depending on the location of the tumour in the stomach.

For a more detailed molecular characterisation of the various CIN-groups, we performed targeted sequencing of a subset of tumours using a GC related gene panel. In line with previous studies in GCs the most frequently mutated gene was the *TP53* gene, which in addition showed a significant association with high degrees of CIN [2, 8]. In contrast, mutations in the *CDH1* gene were almost exclusively found in the CIN-low group. Thus, all these findings are essentially in line with several studies linking *TP53* mutation particularly to structural chromosomal aberrations and *CDH1* mutation to genomic stable tumours, which most likely correspond to our CIN-low group [1, 2, 47].

Interestingly the CIN-substantial group was associated with a higher prevalence of genes involved in the PI3K/AKT signalling pathway such as *PIK3CA*, *PIK3R1* and ERBB family of genes as well as genes involved in cell cycle processes such as *CCND1* and *CDKN2A* which is similar to the findings of the TCGA study for the CIN-subtype [2]. Thus, it is tempting to speculate that particularly patients with tumours of the CIN-substantial group might benefit from treatment with specific inhibitors targeting genes involved in these pathways and several clinical trials evaluating drugs targeting as for example the PI3K/AKT signalling in various tumours are ongoing [48, 49]. In addition, our findings of different mutation patterns in the particular CIN-groups underline the genetic diversity of these groups and support the application of a refined CIN classification.

Our study has limitations, which are the retrospective nature of analyses and thus the inclusion of patients that have not been treated in the frame of a randomised clinical trial. Nevertheless, using a relatively simple method for the determination of CIN applicable in a routine clinical setting and in an analysis of more than 600 tumour samples, our results shed some light on a potentially diverse clinical value of CIN as a biomarker. We are aware that our study has to be considered as an explorative analysis and confirmation of the results in independent studies is mandatory.

In conclusion, our study showed that a more detailed CIN classification might delineate patient groups with different prognostic and predictive impact in GC. Of note, the substantial CIN-level corresponding to an AI ratio in the range of 50–75% in the resected tumours was associated with decreased survival of patients in particular after treatment with neoadjuvant CTx and a high CIN-level corresponding to an AI ratio ≥75% was associated with good response to neoadjuvant CTx only in terms of measurable tumour regression, which however did not directly translate into a considerable survival benefit of the patients. Prospective comprehensive studies are important to confirm our results and in-depth analysis of the tumour microenvironment could broaden our understanding of the biological processes of diverse ‘just-right’ CIN levels in the respective context.

## Supplementary information

Supplementary Material

Reproducibility check list

## Data Availability

All data generated or analysed during this study are included in this published article [and its Supplementary information files].
