# Supplementary material for: Diverse ‘just-right’ levels of chromosomal instability and their clinical implications in neoadjuvant treated gastric cancer
Source: Br J Cancer. 2021 Oct 20;125(12):1621–31. doi: 10.1038/s41416-021-01587-4 (PMC8651679; doi:10.1038/s41416-021-01587-4)
Supplement: Supplementary file 1 — Supplementary Material [file 41416_2021_1587_MOESM1_ESM.pdf]

Supplementary Information

Diverse "just-right" levels of chromosomal instability and their clinical implications in neoadjuvant treated gastric cancer

Meike Kohlruss, Marie Krenauer, Bianca Grosser, Nicole Pfarr, Moritz Jesinghaus, Julia Slotta-Huspenina, Alexander Novotny, Alexander Hapfelmeier, Thomas Schmidt, Katja Steiger, Matthias M. Gaida, Magdalena Reiche, Lukas Bauer, Katja Ott, Wilko Weichert & Gisela Keller

Supplementary Methods

DNA Isolation

DNA from paired tumour and non-tumorous formal-fixed paraffin-embedded (FFPE) tissues was isolated after microdissection from 8µm thick sections after deparaffinization and proteinase K digestions using the Maxwell extraction system according to the instructions of the manufacturer (Promega, Madison, WI, USA) or using a FFPE DNA purification kit (Qiagen, Hilden, Germany). The tumour areas for DNA isolation were marked by experienced pathologists (M.J., J.S.-H.). Tissue samples with only small amounts were re-suspended after manual microdissection in 200 µl of 50 mmol/L Tris-HCl pH 8.5, 1 mmol/L of EDTA, 0.5% of Tween 20, 0.2 mg/ml of proteinase K and incubated at 55°C for 3 hours. Proteinase K was inactivated by boiling for 10 minutes and a 1:3 – 1:10 dilution was directly used for PCR. Non-tumorous and tumour DNA concentrations were determined by measuring the absorbance at 260 nm using a NanoDrop spectrophotometer (Thermo Fisher Scientific, Waltham, MA, USA) or the Qubit DNA quantitation assay (Thermo Fisher Scientific).

Analysis for microsatellite instability (MSI) using mono- and dinucleotide repeats

MSI was analysed using the five markers BAT25, BAT26, D2S123, D5S346 and D17S250 recommended by the National Cancer Institute.[1] A multiplex PCR with fluorescence-tagged primers was performed on non-tumorous and tumour DNA using the Type-it Microsatellite PCR kit according to the instructions of the manufacturer (Qiagen, Hilden, Germany). Tumours that showed instabilities exclusively at dinucleotide repeats were additionally analysed using the three mononucleotide repeat markers NR-21, NR-24 and NR-27 as described.[2] If no instabilities were observed at these mononucleotide repeats, the tumours were reclassified as MSI-L. This additional analysis leads to the reclassification of three resected tumours initially classified as MSI-H to MSI-L.[2]

Cycle conditions of microsatellite based multiplex PCRs and fragment analysis

The cycle conditions were as follows: after an initial step of 95°C for 5 min, 32 cycles were performed consisting of denaturation at 95°C for 30 sec, annealing at 58°C for 90 sec and extension at 72°C for 30 sec and final extension at 60°C for 30 min. Separation and detection of the PCR products were performed in a 3130 Genetic Analyzer (Applied Biosystems, Foster City, CA, USA) loaded with POP-7 polymer (Applied Biosystems) and using ROX-500 Genescan (Thermo Fisher Scientific) as size standard. Samples were analysed with the GeneMapper Software 5 (Applied Biosystems).

Statistical analysis

Comparison between the AI ratios determined by the microsatellite based multiplex PCR assays and the tumour cell contents was performed using the Pearson correlation coefficient (*r*). For the statistical analysis regarding OS and clinical characteristics of the patients, the following tests were used: Kaplan-Meier estimates of survival probabilities were compared by log rank tests. Relative risks were estimated by hazard ratios (HRs) from univariable Cox proportional hazard models. Two-sided Chi-squared tests or Fisher's exact tests were used for hypothesis testing of differences between the relative frequencies. Multivariable analysis was performed by stepwise forward variable selection using Wald-tests of pre- and post-therapeutically available clinical factors and the CIN status was also included as a variable in the model. The pre-therapeutic clinical factors included sex, age (continuous variable), histological Laurén subtypes (intestinal versus non-intestinal), tumour localization (proximal versus non-proximal) and clinical tumour stage (cT2 versus cT3/cT4). The post-therapeutic clinical factors included sex, age (continuous variable), histological Laurén subtypes (intestinal versus non-intestinal), tumour localization (proximal versus non-proximal), (y)pT (pT1-pT4), (y)pN, M-category and R-category. Overall, exploratory 5% significance levels (two-tailed) were used for hypothesis testing. All statistical analyses were performed using SPSS Statistics 25 (IBM corp., Armonk, NY, USA).

Supplementary Figures

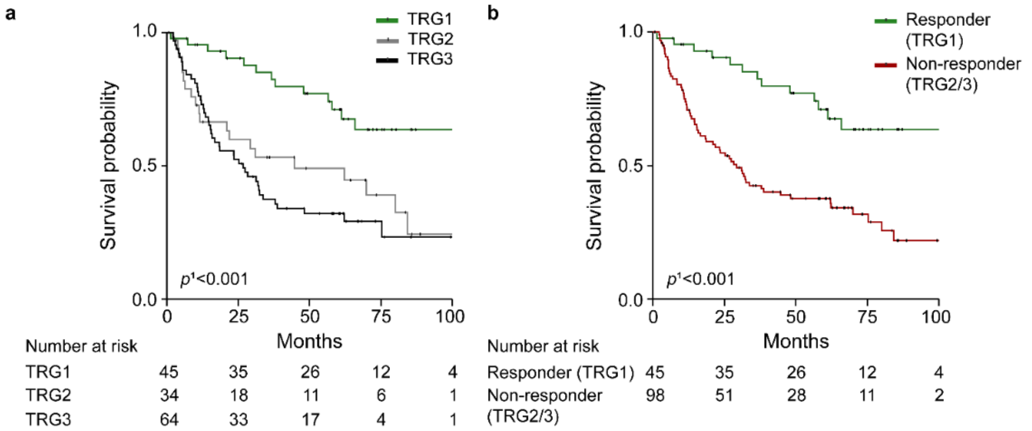

Supplementary Figure S1: Response to neoadjuvant CTx and association with overall survival in the tumour biopsy cohort before CTx (n=143). Patients' survival is discriminated by the three tumour regression grades (TRG1, TRG2, TRG3) (a) and by responders (TRG1) and non-responders (TRG2/3) respectively (b). [1]log-rank test

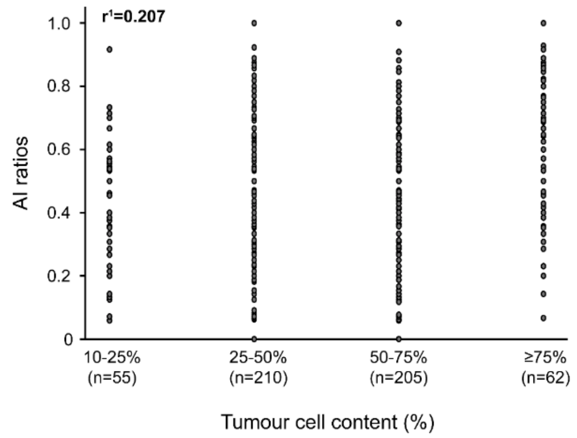

Supplementary Figure S2: Correlation of tumour cell contents and AI ratios of the resected tumours with or without CTx analysed for the refined CIN classification (n=532). AI, allelic imbalance; CIN, chromosomal instability; [1]Pearson correlation coefficient

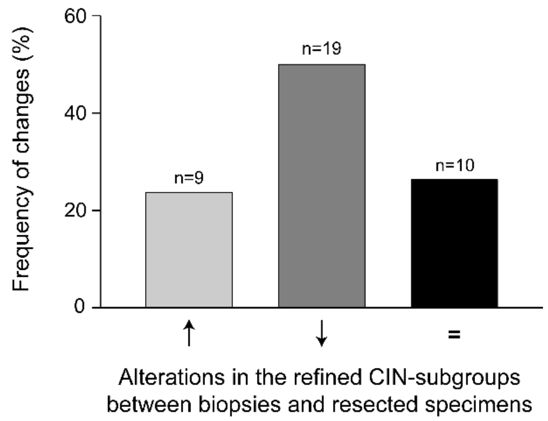

Supplementary Figure S3. Alterations in the four refined CIN-subgroups between corresponding biopsies before and resected tumours after CTx. Change from lower CIN-group in the biopsies before CTx to higher one in the corresponding resected tumours after CTx (↑); change from higher CIN-group in the tumour biopsies to lower one in the corresponding resected tumours (↓); identical CIN classification between tumour biopsies and resected tumours (=). CIN, chromosomal instability

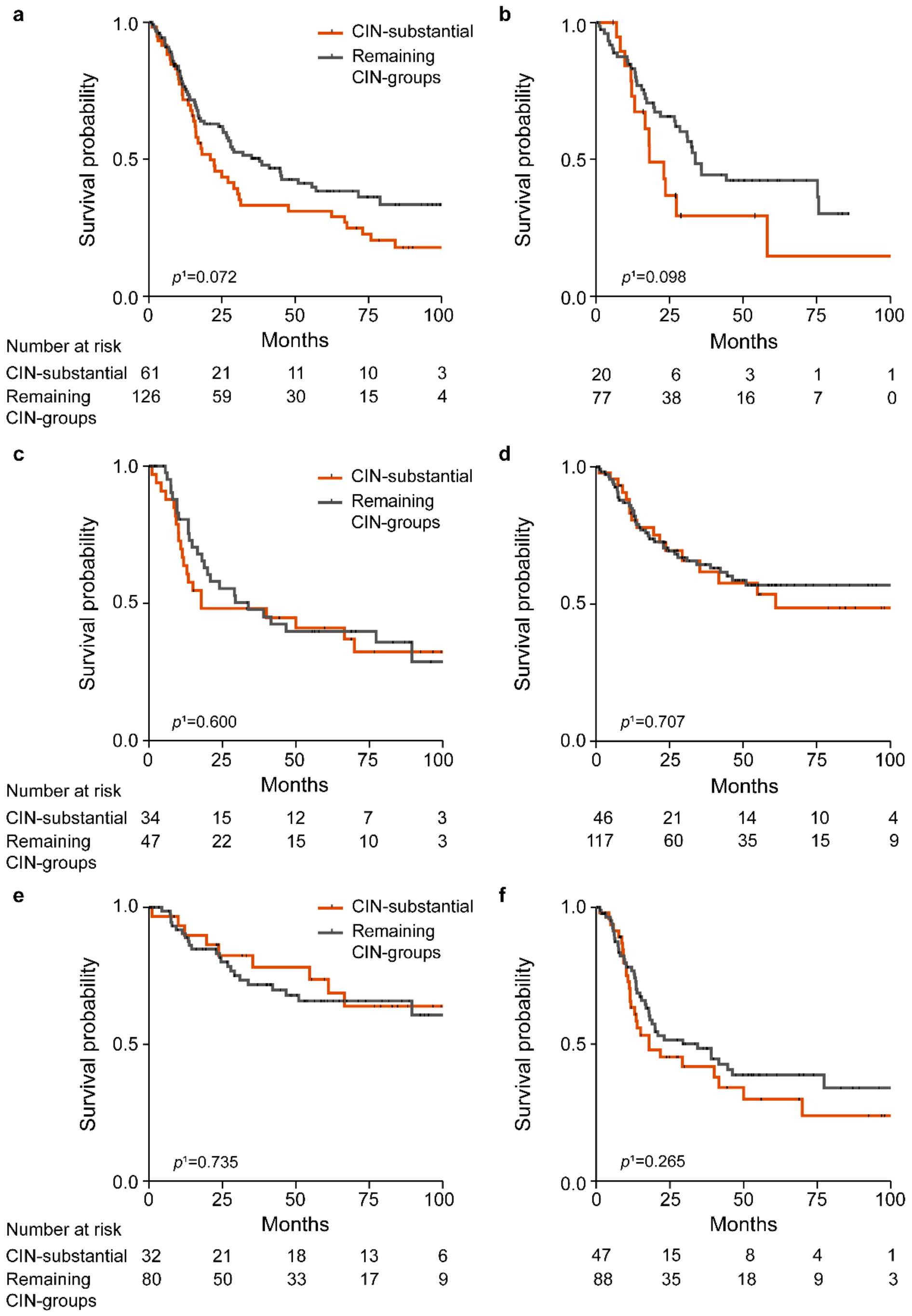

Supplementary Figure S4. Two-tiered CIN classification and association with patients' survival stratified according to tumour localization and clinical tumour stage. Kaplan-Meier curves are shown for patients with resected tumours with CTx harbouring proximal located tumours (a) and non-proximal located tumours (b), for patients with resected tumours without CTx harbouring proximal located tumours (c) and non-proximal located tumours (d) and for patients with resected tumours without CTx with clinical tumour stage cT2 (e) and cT3/4 (f). AI, allelic imbalance; CIN, chromosomal instability; [1]log-rank test

Supplementary Tables

Supplementary Table S1. Neoadjuvant chemotherapy regimens of the patients included for the refined CIN classification

**Neoadjuvant chemotherapy**	**Resected tumours after CTx**		**Tumour biopsies before CTx**	
	n	**%**	***n***	**%**
Total	284	100	122	100
Cis + 5-FU or Cap	113	39.8	101	82.8
Ox + 5-FU or Cap	39	13.7	15	12.3
Cis + 5-FU + Doc or Pac	25	8.8	1	<1
Ox + 5-FU + Doc	16	5.6	0	0
Cis or Ox + 5-FU or Cap + Epi	71	25	4	3.3
Others	19	6.7	1	<1
n/a	1	<1	0	0

Cis, cisplatin; Ox, oxaliplatin; 5-FU, 5-fluorouracil; Cap, capecitabine; Doc, docetaxel; Pac, paclitaxel; Epi, epirubicin; Others, combination of Cis/Ox with other agents as small molecule inhibitors or monoclonal antibodies (Imatinib, Panitumumab, Cetuximab) or cross over between different treatment regimens; No patient received preoperative radiotherapy; n/a, no data available.

Supplementary Table S2. Chromosomal regions covered by microsatellite markers included in the multiplex PCR assays for the detection of AI and CIN

**Chromosomal region**	**Microsatellite marker**
2p21	D2S123[a]
4q22	D4S423
5q11	D5S624
5q21	D5S346[a]
6p25	D6S1617
7q21	D7S492
7q31	D7S486
8p23	D8S552
8q24[b]	D8S1720
8q24[b]	D8S1793
9p21[b]	D9S171
9p21[b]	D9S157
12p12[b]	D12S1682
12p12[b]	D12S1631
16q23	D16S507
17p13	D17S796
17q12[b]	D17S1861
17q12[b]	D17S1872
17q21	D17S250[a]
18q21[b]	D18S1119
18q21[b]	D18S487
19q12	D19S875

[a]Dinucleotide markers included in Bethesda panel for MSI analysis. [b]Chromosomal regions covered with two microsatellite markers.

Supplementary Table S3. Frequency of the molecular subgroups according to TCGA and the refined CIN-groups

	Resected tumour cohorts			Tumour biopsy cohort before CTx n (%)
	All n (%)	With CTx n (%)	Without CTx n (%)	
**Molecular classification according to TCGA**				
EBV(+)	24 (3.9)	16 (5)	8 (2.8)	6 (4.2)
MSI-H	56[a] (9.2)	21[a] (6.5)	35 (12)	15 (10.5)
GS	56 (9.2)	30 (9.3)	26 (8.9)	7 (4.9)
CIN	476 (77.7)	254 (79.2)	222 (76.3)	115 (80.4)
Total	612[b] (100)	321[b] (100)	291 (100)	143 (100)
**Refined CIN classification**				
CIN-low	97 (18.2)	51 (18)	46 (18.5)	10 (8.2)
CIN-moderate	214 (40.2)	121 (42.6)	93 (37.5)	40 (32.8)
CIN-substantial	161 (30.3)	81 (28.5)	80 (32.3)	48 (39.3)
CIN-high	60 (11.3)	31 (10.9)	29 (11.7)	24 (19.7)
Total[c]	532 (100)	284 (100)	248 (100)	122 (100)

MSI-H, high microsatellite instability; GS, genomic stable; CIN, chromosomal instability; [a]Three resected tumours with instabilities only at dinucleotide repeats and classified initially as MSI-H were reclassified as MSS/MSI-L as specified[2]; [b]From five resected tumours of initially included 617 tumours, no CIN data were available. [c]Only EBV and MSI-H negative tumours were analysed for CIN.

Supplementary Table S4. Refined CIN classification and association with clinical characteristics of the resected tumour cohort with CTx (n=284)

**Category**	**Value**	**CIN low *n***	**CIN moderate *n***	**CIN substantial *n***	**CIN high *n***	***p*-value[a]**
**Cases**	Total	51	121	81	31	
**Age Median [yr]**	<64.3	29	69	52	16	0.604
	≥64.3	22	52	29	15	
**Sex**	Male	38	90	70	28	0.061
	Female	13	31	11	3	
**Tumour localization**	Proximal	27	76	61	23	0.037
	Non-proximal	24	45	20	8	
**Laurén classification**	Intestinal	22	64	53	26	0.001
	Non-intestinal	29	57	28	5	
**Tumour grade**	G1/2	9	18	8	7	0.459
	G3/4	31	68	50	18	
	n/a	11	35	23	6	
**Clinical tumour stage**	cT2	3	5	6	0	0.435
	cT3/4	48	116	75	30	
	n/a	-	-	-	1	
**ypT[b]**	ypT1/2	7	15	9	7	0.435
	ypT3/4	44	106	72	24	
**ypN[b]**	Negative	16	35	12	8	0.086
	Positive	35	86	69	23	
**Metastasis status**	No	44	96	63	21	0.256
	Yes	7	25	18	10	
**Resection category**	R0	40	81	59	21	0.394
	R1	11	41	22	10	
**Tumour regression grade**	TRG2	29	56	44	12	0.284
	TRG3	22	65	37	19	

AI, allelic imbalance; CTx, chemotherapy; CIN, chromosomal instability; n/a, not available; TRG, tumour regression grade; [a]Chi-squared or Fisher's exact test; [b]Classification according to 7th Edition UICC 2007.

Supplementary Table S5. Refined CIN classification and association with clinical characteristics of the resected tumour cohort without CTx (n=248)

**Category**	**Value**	**CIN low *n***	**CIN moderate *n***	**CIN substantial *n***	**CIN high *n***	***p*-value[a]**
**Cases**	Total	46	93	80	29	
**Age Median [yr]**	<64.3	19	44	27	9	0.223
	≥64.3	27	49	53	20	
**Gender**	Male	30	62	54	19	0.994
	Female	16	31	26	10	
**Tumour localization**	Proximal	3	30	34	14	<0.001
	Non-proximal	43	60	46	14	
	n/a	-	3	-	-	
**Laurén classification**	Intestinal	7	47	51	22	<0.001
	Non-intestinal	39	46	29	7	
**Tumour grade**	G1/2	4	23	33	11	0.001
	G3/4	42	69	47	18	
	n/a	-	1	-	-	
**Clinical tumour stage**	cT2	30	40	32	10	0.022
	cT3/4	16	53	47	19	
	n/a	-	-	1	-	
**pT[b]**	pT1/2	18	31	24	7	0.551
	pT3/4	28	62	56	22	
**pN[b]**	Negative	17	36	26	8	0.669
	Positive	29	57	54	21	
**Metastasis status**	No	40	87	74	28	0.506
	Yes	6	6	6	1	
**Resection category**	R0	40	68	64	26	0.122
	R1	6	25	16	3	

AI, allelic imbalance; CTx, chemotherapy; CIN, chromosomal instability; n/a, not available; [a]Chi-squared or Fisher's exact test; [b]Classification according to 7th Edition UICC 2007.

Supplementary Table S6. Survival data of the patients with resected tumours with and without CTx in association with the refined CIN classification in four or two subgroups

**Resected tumours with CTx (n=284)**								
**Refined CIN classification**	**No.**	**Events**	**Survival probability [%]**			**Median survival [months]**	**HR**	***p*-value[a]**
			**1 yr**	**3 yr**	**5 yr**	**(95% CI)**	**(95% CI)**	
								0.097
CIN-low	51	23	82.5	43.5	38.7	31.1 (22.0-40.3)	1 ref.	-
CIN-moderate	121	65	78.7	48.5	38.0	35.1 (21.1-49.1)	1.08 (0.67-1.75)	0.741
CIN-substantial	81	55	73.6	32.0	28.6	21.0 (15.5-36.5)	1.53 (0.94-2.49)	0.087
CIN-high	31	13	74.8	54.0	48.6	38.7 (-)	0.84 (0.42-1.66)	0.611
CIN-substantial	81	55	73.6	32.0	28.6	21.0 (15.5-26.5)	1.49 (1.07-2.08)	0.017
Remaining CIN-groups	203	101	79.1	48.4	39.8	35.1 (23.9-46.3)	1 ref.	
Total	284	156	77.5	43.7	36.6	30.3 (25.2-35.4)	-	-
**Resected tumours without CTx (n=248)**								
**Refined CIN classification**	**No.**	**Events**	**Survival probability [%]**			**Median survival [months]**	**HR**	***p*-value[a]**
			**1 yr**	**3 yr**	**5 yr**	**(95% CI)**	**(95% CI)**	
								0.379
CIN-low	46	14	87.7	73.3	58.7	nr	1 ref.	-
CIN-moderate	93	39	81.9	56.5	51.6	77.3 (25.9-128.7)	1.46 (0.79-2.69)	0.227
CIN-substantial	80	40	74.4	56.2	48.3	54.8 (23.7-85.9)	1.69 (0.92-3.11)	0.091
CIN-high	29	15	82.1	52.3	45.7	51.0 (0.96-101.04)	1.68 (0.81-3.47)	0.165
CIN-substantial	80	40	74.4	56.2	48.3	54.8 (23.7-85.9)	1.24 (0.84-1.83)	0.288
Remaining CIN-groups	168	68	83.4	59.9	52.3	89.5 (40.6-138.4)	1 ref.	
Total	248	108	80.5	58.7	50.8	61.1 (27.5-94.7)	-	-

CIN; chromosomal instability; CTx, neoadjuvant chemotherapy; No., number of patients; CI, confidence interval; HR, Hazard ratio; nr, not reached; ref., reference. [a]Cox's regression compared to reference.

Supplementary Table S7. Multivariable analysis of survival including the two-tiered CIN status and pre- and post-therapeutically available clinical factors in the resected tumour cohort with CTx

	HR	95% CI	*p*-value[a]
**Resected tumours with CTx: Pretherapeutic factors**			
**CIN status**			
CIN-substantial	1.48	1.07-2.06	0.019
Remaining CIN-groups	1 ref.	-	
**Resected tumours with CTx: Posttherapeutic factors**			
**R-category**			
R0	0.54	0.38-0.78	0.001
R1	1 ref.	-	
**ypN[b]**			
ypN0	0.40	0.26-0.63	<0.001
ypN1	1 ref.	-	
**ypT[b]**			0.003
ypT1	0.43	0.10-1.81	0.252
ypT2	0.43	0.21-0.88	0.021
ypT3	0.53	0.37-0.75	<0.001
ypT4	1 ref.	-	-
**Metastasis status**			
Negative	0.56	0.39-0.81	0.002
Positive	1 ref.	-	
**CIN status**			
CIN-substantial	1.48	1.05-2.08	0.026
Remaining CIN-groups	1 ref.	-	

ref., reference; CIN, chromosomal instability; HR; Hazard ratio; CI, confidence interval. [a]Wald-Test of Hazard Ratio; [b]TNM classification according to 7th Edition UICC

Supplementary Table S8. Multivariable analysis of survival including the two-tiered CIN status and pre- and post-therapeutically available clinical factors in the resected tumour cohort without CTx

	HR	95% CI	*p*-value[a]
**Resected tumours without CTx: Pretherapeutic factors**			
**cT**			
cT2	0.34	0.23-0.52	<0.001
cT3/4	1 ref.	-	
**Age**	1.03	1.01-1.05	<0.001
**Resected tumours without CTx: Posttherapeutic factors**			
**pN[b]**			
pN0	0.27	0.16-0.46	<0.001
pN1	1 ref.	-	
**Age**	1.03	1.01-1.04	0.002
**Metastasis status**			
Negative	0.40	0.20-0.80	0.010
Positive	1 ref.	-	
**Localization**			
Proximal	1.56	1.06-2.30	0.025
Non-proximal	1 ref.	-	

ref., reference; CIN, chromosomal instability; HR; Hazard ratio; CI, confidence interval. [a]Wald-Test of Hazard Ratio; [b]TNM classification according to 7th Edition UICC

Supplementary Table S9. Survival data of the patients with tumour biopsies before CTx in association with the refined CIN classification and response to neoadjuvant CTx

**Refined CIN classification**	**No.**	**Events**	**Survival probability [%]**			**Median survival [months]**	**HR**	***p*-value[a]**
			**1 yr**	**3 yr**	**5 yr**	**(95% CI)**	**(95% CI)**	
								0.674
CIN-low	10	5	90.0	64.3	64.3	62.1 (4.7-119.5)	1 ref.	-
CIN-moderate	40	24	73.2	47.9	44.7	33.8 (4.3-63.3)	1.35 (0.51-3.54)	0.546
CIN-substantial	48	24	75.0	55.1	50.5	62.2 (-)	0.98 (0.37-2.59)	0.973
CIN-high	24	15	73.9	55.9	37.3	37.9 (27.0-48.8)	1.32 (0.48-3.64)	0.592
								0.004
CIN-high, responder	12	6	81.8	63.6	45.5	57.8 (-)	0.62 (0.26-1.44)	0.226
CIN-high, non-responder	12	9	66.7	48.6	29.2	31.1 (1.9-60.2)	1.10 (0.54-2.26)	0.786
non-CIN-high, responder	26	5	100	90.9	86.1	nr	0.19 (0.08-0.48)	<0.001
non-CIN-high, non-responder	72	48	67.5	40.7	37.2	27.4 (16.7-38.1)	1 ref.	-
Total	122	68	75.6	54.1	47.1	44.6 (18.5-70.8)	-	-

CIN; chromosomal instability; CTx, neoadjuvant chemotherapy; No., number of patients; CI, confidence interval; HR, Hazard ratio; nr, not reached; ref., reference. [a]Cox's regression compared to reference.

Supplementary Table S10. Sequence variants identified by targeted sequencing using a custom designed GC related gene panel

Gene	Exon	Ref Seq Number	cDNA description	Protein description	Type of mutation	Entry in database[a]
APC	17	NM_001127510.3	c.4348C>T	p.R1450*	Nonsense	COSV57321313
ARID1A	5	NM_006015.6	c.2077C>T	p.R693*	Nonsense	COSV61375361
ARID1A	20	NM_006015.6	c.5965C>T	p.R1989*	Nonsense	COSV61370535
ARID1A	20	NM_006015.6	c.6020_6036del	p.L2007Qfs*20	Frameshift deletion	-
ATM	29	NM_000051.4	c.4385C>G	A1462G	Missense	-
CCND1	5	NM_053056.3	c.775_790del	p.L259Rfs*91	Frameshift deletion	-
CDH1	1	NM_004360.5	c.11G>A	p.W4*	Nonsense	-
CDH1	3	NM_004360.5	c.220C>T	p.R74*	Nonsense	COSV55732282
CDH1	5	NM_004360.5	c.641T>C	p.L214P	Missense	COSV55734778
CDH1	7	NM_004360.5	c.948_956del	p.M316_T318del	Nonframeshift deletion	-
CDH1	7	NM_004360.5	c.975_998del	p.I326_L333del	Nonframeshift deletion	-
CDH1	8	NM_004360.5	c.1009-2A>G	p.?	Splice acceptor variant	cBioPortal
CDH1	8	NM_004360.5	c.1088T>A	p.I363N	Missense	-
CDH1	10	NM_004360.5	c.1765-1_1765-4del	p.?	Splice region variant	-
CDH1	13	NM_004360.5	c.2145_2164del	p.G716Sfs*25	Frameshift deletion	-
CDKN2A	1	NM_058197.4	c.148_164del	p.Q50Cfs*23	Frameshift del.	-
CDKN2A	2	NM_000077.5	c.160_162del	p.M54del	Nonframeshift deletion	COSV58699795
CTNNA1	6	NM_001903.5	c.643C>T	p.Q215*	Nonsense	-
CTNNB1	3	NM_001904.4	c.134C>T	p.S45F	Missense	COSV62687872
ERBB2	8	NM_004448.4	c.929C>T	p.S310F	Missense	COSV54062198
ERBB2	20	NM_004448.4	c.2329G>C	p.V777L	Missense	COSV54062385
ERBB3	7	NM_001982.4	c.734C>T	p.A245V	Missense	COSV57248597
ERBB3	7	NM_001982.4	c.850G>A	p.G284R	Missense	COSV57246350
ERBB4	23	NM_005235.3	c.2762A>T	p.Y921F	Missense	-
FBXW7	9	NM_033632.3	c.1322G>A	p.R441Q	Missense	COSV55902900
FBXW7	10	NM_033632.3	c.1513C>T	p.R505C	Missense	COSV55891274
FGFR1	8	NM_023110.3	c.1010G>A	p.G337E	Missense	rs1064793122
FGFR1	8	NM_023110.3	c.1042G>A	p.G348R	Missense	rs886037634
FGFR1	10	NM_023110.3	c.1334G>A	p.R445Q	Missense	-
KRAS	2	NM_033360.4	c.34G>A	p.G12S	Missense	COSV55497461
KRAS	2	NM_033360.4	c.35G>T	p.G12V	Missense	COSV55497419
NRAS	2	NM_002524.5	c.34G>T	p.G12C	Missense	COSV54736487
PIK3CA	10	NM_006218.4	c.1624G>A	p.E542K	Missense	COSV55873227
PIK3CA	10	NM_006218.4	c.1634A>C	p.E545A	Missense	COSV55873209
PIK3CA	10	NM_006218.4	c.1633G>A	p.E545K	Missense	COSV55873239
PIK3R1	13	NM_181523.3	c.1709_1714delinsGGA	p.L570_Q572delinsRK	Nonframeshift substitution	-
PREX2	2	NM_024870.4	c.148T>G	p.L50V	Missense	COSV55751655
PREX2	2	NM_024870.4	c.190A>C	p.N64H	Missense	-
PREX2	24	NM_024870.4	c.2738G>T	p.R913M	Missense	COSV99909747
PTEN	5	NM_000314.8	c.335T>A	p.L112Q	Missense	COSV64304040
PTPRT	8	NM_133170.4	c.1317G>T	p.E439D	Missense	COSV61984828
RHOA	2	NM_001664.4	c.125A>G	p.Y42C	Missense	COSV69041523
RNF43	8	NM_017763.6	c.935G>A	p.C312Y	Missense	-
SMAD4	9	NM_005359.6	c.1049T>G	p.V350G	Missense	COSV61684103
SMAD4	9	NM_005359.6	c.1067C>A	p.P356H	Missense	COSV61689602
TGFBR2	5	NM_001024847.2	c.1243G>A	p.D415N	Missense	COSV55447128
TGFBR2	5	NM_001024847.2	c.1228_1235dup	p.L413Sfs*3	Frameshift insertion	-
TGFBR2	5	NM_001024847.2	c.1259T>A	p.L420Q	Missense	-
TGFBR2	5	NM_001024847.2	c.1217A>G	p.K406R	Missense	-
TGFBR2	6	NM_001024847.2	c.1406_1409del	p.Q469Pfs*13	Frameshift deletion	-
TGFBR2	8	NM_001024847.2	c.1649C>G	p.P550R	Missense	-
TLR4	3	NM_138554.5	c.1459T>G	p.F487V	Missense	COSV62923063
TP53	4	NM_000546.5	c.338dup	p.H115Afs*34	Frameshift insertion	COSV53530722
TP53	5	NM_000546.5	c.524G>A	p.R175H	Missense	COSV52661038
TP53	5	NM_000546.5	c.541C>T	p.R181C	Missense	COSV52689134

TP53	5	NM_000546.5	c.404G>A	p.C135Y	Missense	COSV52675774
TP53	5	NM_000546.5	c.527G>A	p.C176Y	Missense	COSV52660760
TP53	5	NM_000546.5	c.392A>T	p.N131I	Missense	COSV52718021
TP53	5	NM_000546.5	c.437G>A	p.W146*	Nonsense	COSV52661900
TP53	6	NM_000546.5	c.567_577del	p.P190S*15	Frameshift deletion	-
TP53	6	NM_000546.5	c.659A>G	p.Y220C	Missense	COSV52661282
TP53	6	NM_000546.5	c.560_562-7del	p.?	Splice region variant	cBioPortal
TP53	7	NM_000546.5	c.764_766del	p.I255del	Nonframeshift deletion	COSV52737696
TP53	7	NM_000546.5	c.743G>A	p.R248Q	Missense	COSV52661580
TP53	7	NM_000546.5	c.722C>T	p.S241F	Missense	COSV52661688
TP53	7	NM_000546.5	c.673-1G>C	p.?	Splice acceptor variant	COSV52707963
TP53	8	NM_000546.5	c.824G>A	p.C275Y	Missense	COSV52661919
TP53	8	NM_000546.5	c.892G>T	p.E298*	Nonsense	COSV52661551
TP53	8	NM_000546.5	c.817C>T	p.R273C	Missense	COSV52662066
TP53	8	NM_000546.5	c.818G>A	p.R273H	Missense	COSV52660980
TP53	8	NM_000546.5	c.818G>T	p.R273L	Missense	COSV52664805
TP53	8	NM_000546.5	c.844C>T	p.R282W	Missense	COSV52662048
TP53	8	NM_000546.5	c.916C>T	p.R306*	Nonsense	COSV52662281
TP53	9	NM_000546.5	c.920-2A>G	p.?	Splice acceptor variant	COSV52706655
TP53	9	NM_000546.5	c.989T>G	p.L330R	Missense	COSV52677086
TP53	10	NM_000546.5	c.1043T>G	p.L348W	Missense	COSV52736234
TP53	10	NM_000546.5	c.1009C>T	p.R337C	Missense	COSV52669243
XIRP2	9	NM _152381.5	c.4131_4133del	p.E1377del	Nonframeshift deletion	COSV54683582

[a]Databases: Catalogue Of Somatic Mutations In Cancer (COSMIC): Genomic Mutation Identifier (COSV)[3], cBioPortal[4,5], NCBI dbSNP database[6].

Supplementary References

1. Boland C.R., Thibodeau S.N., Hamilton S.R., Sidransky D., Eshleman J.R., Burt R.W. et al. A National Cancer Institute Workshop on Microsatellite Instability for cancer detection and familial predisposition: development of international criteria for the determination of microsatellite instability in colorectal cancer. *Cancer Res* **58**, 5248-5257 (1998).

2. Kohlruss M., Ott K., Grosser B., Jesinghaus M., Slotta-Huspenina J., Novotny A. et al. Sexual Difference Matters: Females with High Microsatellite Instability Show Increased Survival after Neoadjuvant Chemotherapy in Gastric Cancer. *Cancers* **13**, 1048 (2021).

3. Tate J.G., Bamford S., Jubb H.C., Sondka Z., Beare D.M., Bindal N. et al. COSMIC: the Catalogue Of Somatic Mutations In Cancer. *Nucleic Acids Res* **47**, D941-d947 (2019).

4. Cerami E., Gao J., Dogrusoz U., Gross B.E., Sumer S.O., Aksoy B.A. et al. The cBio cancer genomics portal: an open platform for exploring multidimensional cancer genomics data. *Cancer Discov* **2**, 401-404 (2012).

5. Gao J., Aksoy B.A., Dogrusoz U., Dresdner G., Gross B., Sumer S.O. et al. Integrative analysis of complex cancer genomics and clinical profiles using the cBioPortal. *Sci Signal* **6**, pl1 (2013).

6. Sherry S.T., Ward M.H., Kholodov M., Baker J., Phan L., Smigielski E.M. et al. dbSNP: the NCBI database of genetic variation. *Nucleic Acids Res* **29**, 308-311 (2001).
